# Are pre-frail and frail amyloid positive individuals eligible to Lecanemab? A cross-sectional analysis from the Cogfrail real-world cohort

**DOI:** 10.1186/s13195-026-01966-0

**Published:** 2026-02-04

**Authors:** Federico Bellelli, Julien Delrieu, Gabor Abellan van Kan, Alberta Peluso, Gaëlle Soriano, Bruno Vellas, Davide Angioni, Sandrine Sourdet

**Affiliations:** 1 IHU HealthAge, Toulouse, France; 2 Institut du Vieillissement, Gérontopôle de Toulouse, Centre Hospitalo-Universitaire de Toulouse, 37 Allées Jules Guesde, Toulouse, 31000 France; 3 CERPOP, Inserm 1295, Toulouse University, INSERM, UPS, Toulouse, France; 4 Alzheimer’s Disease Research and Clinical Center, Toulouse University Hospital, Toulouse, 31000 France; 5 Maintain Aging Research Team, CERPOP, Université de Toulouse, INSERM, Université Paul Sabatier, Toulouse, France; 6 Gérontopôle, Department of Geriatrics, Toulouse University Hospital, La Cité de La Santé, Hôpital La Grave, Toulouse, France

**Keywords:** Lecanemab, Clarity-AD, Frailty, Alzheimer’s disease, Amyloid, Older adults, Real-world cohort

## Abstract

**Background:**

Following the positive outcomes of the Clarity-AD trial, Lecanemab received marketing authorization from the European Medicines Agency (EMA) and is expected to become available across Europe. However, the trial did not specifically evaluate frailty, making it difficult to estimate the potential effects of Lecanemab among frail individuals. This study aimed to apply Lecanemab eligibility criteria—based on both the Clarity-AD trial and the Appropriate Use Recommendations (AUR) from the United States and France—to a real-world population of pre-frail and frail older adults with confirmed positive amyloid status, and to evaluate differences in frailty status between eligible and non-eligible patients.

**Methods:**

Eligibility criteria from the Clarity-AD trial, the American and the French AUR, were applied to all participants with confirmed amyloid positivity (*n* = 120), assessed through amyloid-PET (visual reading) or cerebrospinal fluid (CSF) analysis (Aβ42 levels or Aβ42/Aβ40 ratio). Frailty was defined using the Fried phenotype.

**Results:**

The median age of the sample was 82.0 years (IQR: 79–85); 65% (*n* = 78) were women, and 36.7% (*n* = 44) were frail. Overall, 20.0% (*n* = 24) met the Clarity-AD eligibility criteria, while 50.8% (*n* = 61) and 47.5% (*n* = 57) were potentially eligible according to the American and French AURs, respectively. Only 9.1% (*n* = 4) of frail individuals met the Clarity-AD criteria, compared to 26.3% (*n* = 20) of pre-frail participants (*p* = 0.042). In contrast, 50.0% (*n* = 22) and 45.5% (*n* = 20) of frail individuals were potentially eligible according to the American and French AURs, respectively.

**Conclusion:**

Although less than one in five participants would have been eligible for the Clarity-AD trial, approximately half the cohort would be potentially treatable with Lecanemab under real-world recommendations. While a considerable proportion of frail patients may have access to Lecanemab treatment in real-life, the low proportion of potentially eligible frail individuals for Clarity-AD in our cohort indirectly suggests that frailty may have been underrepresented in the trial, raising concerns about the generalizability of its findings to this population. Caution is warranted when targeting amyloid burden without previously addressing the underlying frailty.

**Trial registration:**

NCT03129269.

**Supplementary Information:**

The online version contains supplementary material available at 10.1186/s13195-026-01966-0.

## Introduction

Alzheimer's disease (AD) is the leading cause of dementia, accounting for approximately 60—70% of the more than 55 million cases worldwide [1]. While the age-specific incidence of dementia has been declining in high-income countries [2], the overall number of affected individuals is expected to nearly triple by the year 2050 due to population aging [1]. Following nearly two decades of intense research and repeated failures in drug development, the therapeutic landscape for AD has entered a new era with the approval of three distinct disease-modifying therapies (DMTs) in the United States [3–5]. In particular, Lecanemab received marketing authorization also from the European Medicines Agency (EMA) in November 2024 and is expected to become available in European countries in the near future [5]. This approval was based on the positive results of the Clarity-AD phase III trial [6], which demonstrated significant efficacy across all clinical outcomes, with nearly a one-third reduction in disease progression on the primary endpoint, the Clinical Dementia Rating Sum of Boxes (CDR-SB).

Although the approval of a DMT for AD represents a major therapeutic advance for millions of older adults affected by the disease, it also raises critical concerns regarding healthcare costs and service delivery. A recent study estimates that, even without accounting for the costs of identifying amyloid-positive patients, treating all potentially eligible patients in Europe with Lecanemab would cost approximately 133 billion euros annually, compared to the total European pharmaceutical expenditures of around 255 billion euros in 2021 [7]. Moreover, Lecanemab treatment will necessitate substantial healthcare infrastructure, as it requires biweekly intravenous infusions and intensive MRI monitoring to detect potential adverse effects, such amyloid-related imaging abnormalities-hemorrhages (ARIA-H) and -edema (ARIA-E) [7].

Frailty is a clinically recognizable condition characterized by reduced physiologic reserve and heightened susceptibility to a wide spectrum of adverse health outcomes [8]. Over the past two decades, the concept of frailty has gained increasing relevance across various settings and has now been incorporated into several guidelines in both medical [9, 10] and surgical fields [11], guiding the development of personalized care plans tailored to an individual’s frailty status. In the context of AD treatment, frailty may serve as a valuable stratification tool, assisting clinicians in selecting appropriate therapeutic intensities based on patient’s capacity to tolerate and benefit from treatment. However, similar to the ENGAGE/EMERGE [12] and TRAILBLAZER-ALZ 2 [13] trials, Clarity-AD did not evaluate frailty among enrolled participants. Moreover, although several studies have applied Clarity-AD eligibility criteria to real-world populations, so far, none have specifically addressed frailty [14–17]. As a result, it remains unclear whether frail individuals would have met the inclusion criteria for the phase III trial, making it difficult to estimate the potential effects of Lecanemab in this specific population.

The aim of this study was to apply Lecanemab eligibility criteria—based on both the Clarity-AD trial and recommendations from the American [18] and French [5] Appropriate Use Recommendations (AUR)—to a real-world population of pre-frail and frail older adults with confirmed positive amyloid status, and to evaluate differences in physical frailty status between eligible and non-eligible patients.

## Methods

### Study design and participants

This is a secondary analysis using baseline data from older adults enrolled in the Cognitive Function and Amyloid Marker in Frail Older Adults, Cogfrail cohort (registration: NCT03129269). Briefly, Cogfrail is a single-center, prospective observational study designed to assess the prevalence of positive cerebral amyloid status among older people with early cognitive impairment, while tracking their cognitive and physical evolution over a 2-year follow-up. The study cohort included community-dwelling older adults who met the following criteria: age ≥ 70 years, Clinical Dementia Rating (CDR) score of 0.5 or 1 (indicative of mild cognitive impairment and mild dementia, respectively), Mini-Mental State Examination (MMSE) ≥ 20, and at least one frailty criterion according to Fried’s Criteria (weakness, slow walking speed, fatigue, unintentional weight loss and low physical activity). Participants were classified as frail if they met ≥ 3 of the 5 criteria (unintentional weight loss, exhaustion, low physical activity, slowness, and weakness), and as pre-frail if they met 1 or 2 criteria [19]. Individuals with severe clinical or psychological conditions that could interfere with study procedures (e.g., active cancer or decompensated psychotic disorders), as well as those with dependence in more than two Activities of Daily Living (ADLs), were not included. Full details on the COGFRAIL study have been published previously [20]. The COGFRAIL study received ethical approval from the institutional research committee (Registration Number: RC31/16/8753; Registration Date: 2017–03-02), and participants provided written informed consent following the Declaration of Helsinki.

Participants were enrolled during routine outpatient visits at the Frailty Clinic or Memory Clinic of the Gérontopôle, Toulouse University Hospital, or during community-care assessments, between January 2017 and February 2020 as part of their routine clinical care. The COGFRAIL study was designed to recruit participants with frailty and cognitive impairment, following referral by their general practitioner, specialist or geriatrician for a standard frailty and/or cognitive assessment, and to ensure a follow-up consistent with the routine care usually provided in our units.

After excluding participants with unavailable amyloid status data and those without evidence of cerebral amyloid burden, the final sample consisted of 120 older adults with confirmed amyloid pathology.

### Amyloid status determination

Amyloid positivity in our sample was assessed using either amyloid positron emission tomography (amyloid-PET) (*n* = 108) or cerebrospinal fluid (CSF) (*n* = 12). Full details about amyloid determination protocols are available elsewhere [20]. In line with current clinical practice, amyloid-PET positivity was determined through visual read analyses conducted by three independent nuclear medicine physicians who were blinded to the participants’ clinical information. A binary classification system was used, categorizing individuals as amyloid-positive or amyloid-negative based on cortical tracer retention. Lumbar puncture for CSF amyloid-β1–42 measurement was proposed as an alternative method in patients who declined the PET scan or had contraindications to the procedure. CSF amyloid quantification was conducted at the Toulouse BioResources Center (Centre de Ressources Biologiques, CRB) using two standardized assays: the enzyme-linked immunosorbent assay (ELISA, INNOTEST, FUJIREBIO) before September 2019, and the chemiluminescent enzyme immunoassay (CLEIA, Lumipulse-G600-II, FUJIREBIO) subsequently. Patients were classified as positive if the CSF-Aβ42 level was < 500 pg/mL for ELISA INNOTEST or < 600 pg/mL for CLEIA. If the CSF-Aβ42 level exceeded the cut-off, the Aβ42/Aβ40 ratio was used to further confirm amyloid status, with thresholds of < 0.05 for ELISA INNOTEST and < 0.07 for CLEIA. Patients were classified as positive if either the Aβ42 level or the Aβ42/Aβ40 ratio were below their respective cut-offs.

### Eligibility criteria for the phase III Clarity-AD trial:

To apply the eligibility criteria of the Clarity-AD trial, two investigators (FB and DA) independently reviewed the medical records retrospectively. In cases of disagreement, a third investigator (JD) was consulted to reach a consensus. Full details on these criteria are provided elsewhere [6]. Briefly, individuals were considered ineligible if they did not meet the following inclusion criteria: age between 50 and 90 years, MMSE score ≥ 22, and Body Mass Index (BMI) between 17 and 35. For Clarity-AD, exclusion criteria were grouped into four categories and applied sequentially. Participants were classified as non-eligible if they met at least one exclusion criterion:

1. Relevant clinical history issues: including psychiatric diagnosis or symptoms, a Geriatric Depression Scale score > 8, unstable medical conditions (e.g., cardiac, respiratory, gastrointestinal, or renal disease that could compromise participant safety or interfere with study assessments) or recent medical events (e.g., history of transient ischemic attacks, stroke, or seizures within the past 12 months).
2. Medication-related issues: individuals not on a stable dose of concomitant medications—at least 12 weeks for AD treatments (e.g., acetylcholinesterase inhibitors or memantine) and at least 4 weeks for other medications. Participants receiving anticoagulation were considered eligible for the Clarity-AD trial if they had been on a stable dose for at least 4 weeks.
3. Other factors: including incompatibility with MRI due to pacemakers, implantable cardioverter-defibrillators (ICDs), or severe claustrophobia.
4. MRI findings leading to exclusion: including macrohemorrhages or more than four microhemorrhages, multiple lacunar infarcts or strokes involving a major vascular territory, superficial siderosis, vasogenic edema, or other major intracranial pathology.

### Eligibility criteria for real-world recommendations:

Considering the great overlap between American and French AUR, the methodological application of both AURs will be discussed together in this section*.* Supplementary Table 1 summarizes the differences between Clarity-AD, American-AUR and French-AUR eligibility criteria. Full details on these criteria are available elsewhere [5, 18]. Briefly, participants were not excluded based only on age, BMI or MMSE scores. Indeed, although the French AUR indicate that an MMSE score ≥ 22 is typically required, they also allow exceptions in specific situations (such as low educational level) and recommend prioritizing the CDR over MMSE to better characterize the severity of cognitive impairment. Exclusion criteria were grouped into four categories and applied sequentially. People were considered non-eligible according to the AURs if they met at least one exclusion criteria:

1. Anticoagulant medication use: participants receiving anticoagulation therapy were considered ineligible.
2. Apolipoprotein-E (Apo-E) E4/E4 homozygosis: people with a known homozygosis for Apo-E E4/E4 were excluded for the French-AUR, while were classified under the 'collegial discussion' category for the American-AUR.
3. Other factors: including incompatibility with MRI due to pacemakers, implantable cardioverter-defibrillators (ICDs), or severe claustrophobia.
4. MRI findings leading to exclusion: including macrohemorrhages or more than four microhemorrhages, multiple lacunar infarcts or strokes involving a major vascular territory, superficial siderosis, vasogenic edema, or other major intracranial pathology. Individuals with less than five microhemorrhages but more than one lobar microhemorrhage—suggestive of probable cerebral amyloid angiopathy (CAA)—were excluded for the French AUR, but not for the American AUR.

Considering that medically unstable conditions may improve with appropriate treatment and given the retrospective nature of our analyses—which limited our ability to ascertain whether these conditions were severe enough to justify exclusion—relevant medical history was included under the 'collegial discussion' category for real-world recommendations. For the American AUR, also individuals aged 90 or above were classified within the “collegial discussion” category.

### Statistical analyses

Participants’ characteristics were summarized using descriptive statistics (median, interquartile range [IQR], count and percentage). To compare groups, the Student's t-test was used for continuous variables, while the chi-square test was applied to categorical variables. Statistical analyses were conducted using the R (Version 2023·12·1 + 402), with a significance level defined at *p <* 0.05.

## Results

**Fig. 1:**
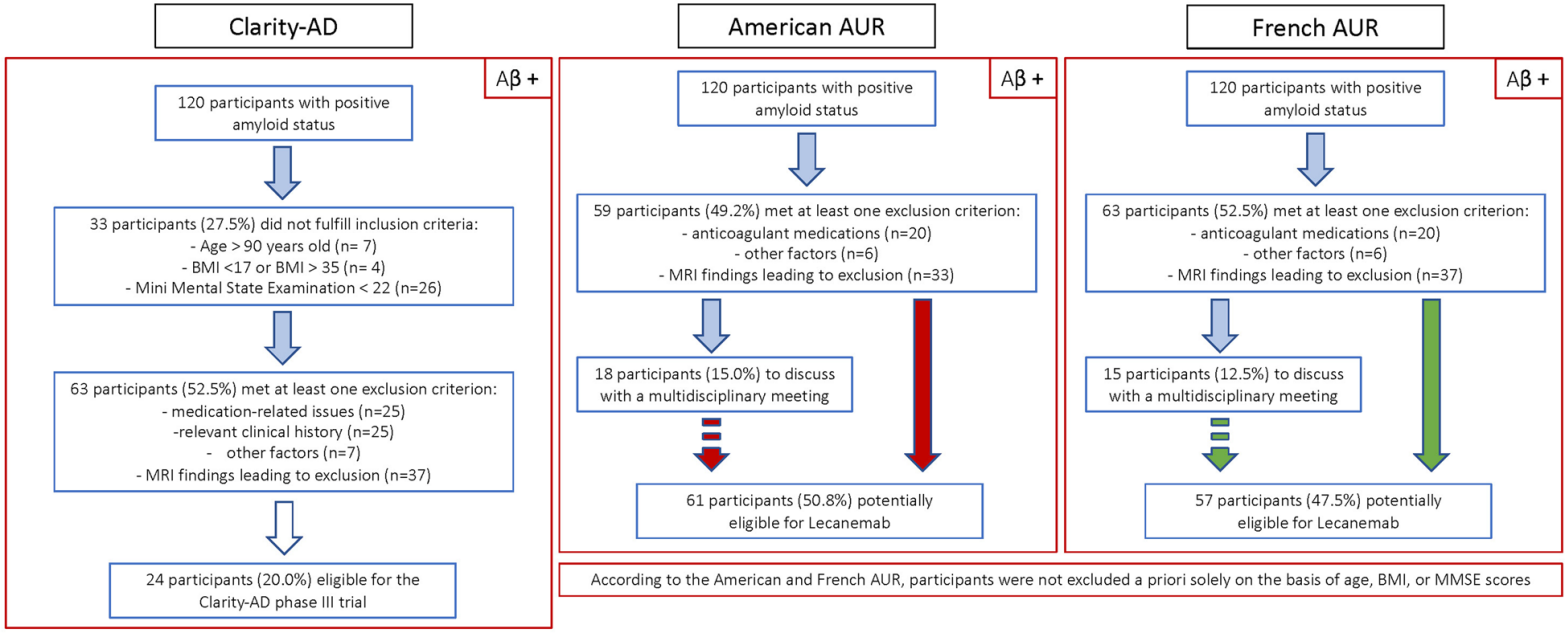
Exclusion flowchart in the Cogfrail population according to phase III Clarity-AD trial eligibility criteria and American and French AUR. *Legend: Aβ, Beta Amyloid; AUR, Appropriate Use Recommendations;* *BMI, Body Mass Index; MMSE, Mini Mental State Examination, MRI, Magnetic Resonance Imaging; n, number of people*

The exclusion process according to the three recommendations is outlined in Fig. 1. Overall, 20.0% (*n* = 24) met the Clarity-AD eligibility criteria, while 50.8% (*n* = 61) and 47.5% (*n* = 57) were potentially eligible according to the American and French AURs, respectively. However, 29.5% (*n* = 18) of those meeting American criteria and 25.8% (*n* = 15) of those meeting French criteria would require a collegial discussion before receiving final approval for treatment. ApoE genotype information was available for 112 participants, of whom only 2 (1.8%) had an ε4/ε4 genotype. Both would have been excluded from real-world Lecanemab treatment due to anticoagulation therapy, regardless of their genotype.

Characteristics of the population according to eligibility status are summarized in Table 1. The median age of the sample was 82.0 (IQR: 79–85) years (age range: 71–93), with 65% (*n* = 78) being women. The median MMSE score was 24 (range: 20–30), and among the 26 patients with an MMSE < 22, twenty (76.9%) had a low level of education. Relevant clinical history leading to exclusion from the Clarity-AD trial or to multidisciplinary discussion under the American and French AURs is detailed in Supplementary Table 2. Based on the frailty phenotype criteria, 63.3% (*n* = 76) of the participants were classified as pre-frail and 36.7% (*n* = 44) as frail. Eligible individuals according to the Clarity-AD criteria had a higher educational level (*p <* 0.001), higher scores on the Short Physical Performance Battery (SPPB) (*p* = 0.002), and faster gait speed (*p* = 0.010) compared to non-eligible participants. No significant differences were observed between eligible and non-eligible individuals based on the American or French recommendations. In addition, only 9.1% (*n* = 4) of frail individuals met the Clarity-AD eligibility criteria, compared to 26.3% (*n* = 20) of pre-frail participants (*p* = 0.042). In contrast, 50.0% (*n* = 22) and 45.5% (*n* = 20) of frail individuals were potentially eligible according to the American and French AURs, respectively, with no significant difference in eligibility rates according to frailty status.

**Table 1:** Characteristics of the population according to eligibility status

		**Clarity-AD Eligibility**			**American AUR Eligibility**			**French AUR Eligibility**		
	Total (*n* = 120)	**Yes (*n* = 24)**	**No (= 96)**	***p* value**	**Yes**^**§**^** (*n* = 61)**	**No (*n* = 59)**	***p* value**	**Yes**^**§**^** (*n* = 57)**	**No (*n* = 63)**	***p* value**
Age, median (IQR)	82.0 (79.0–85.0)	82.0 (78.8–84.2)	82.5 (79.0–86.0)	0.212	82.0 (79.0–85.0)	82.0 (79.5–86.0)	0.544	82.0 (79.0–85.0)	82.2 (79.0–85.5)	0.871
Sex (Female), n (%)	78 (65.0%)	14 (58.3%)	64 (66.7%)	0.598	44 (72%)	34 (57.6%)	0.141	40 (70.2%)	38 (60.3%)	0.348
Living situation: Alone (vs other), n (%)	40 (33.3%)	9 (37.5%)	31 (32.3%)	0.808	20 (32.7%)	20 (33.9%)	1.000	19 (33.3%)	21 (33.3%)	1.000
Education: High (vs Low), n (%)	33 (42.8%)	18 (75.0%)	33 (34.7%)	< 0.001	28 (46.7%)	23 (38.9%)	0.508	26 (46.4%)	25 (39.7%)	0.577
BMI, median (IQR)	25.2 (22.7–28.2)	24.8 (22.3–27.3)	25.2 (22.9–28.4)	0.710	25.1 (22.7–28.6)	25.3 (22.8–28.0)	0.808	24.9 (22.6–28.6)	25.3 (22.8–28.0)	0.953
FRIED score, n (%)										
Pre-frail	76 (63.3%)	20 (26.3%)	56 (73.7%)	0.004	39 (51.3%)	37 (48.7%)	1.000	37 (48.7%)	39 (51.3%)	0.879
Frail	44 (36.7%)	4 (9.1%)	40 (90.1%)		22 (50.0%)	22 (50.0%)		20 (45.5%)	24 (54.5%)	
Hand grip strength, kg, median (IQR)	19.0 (14.0–24.0)	20.0 (16.0–29.0)	18.0 (14.0–24.0)	0.088*	19.0 (14.0–24.0)	20.0 (15.0–24.5)	0.895	19.0 (14.0–24.0)	20.0 (15.0–24.0)	0.977
Gait speed (4 m), median (IQR)	0.83 (0.69–0.99)	0.94 (0.78–1.08)	0.82 (0.66–0.95)	0.010	0.84 (0.70–1.02)	0.83 (0.68–0.95)	0.259	0.84 (0.69–1.02)	0.83 (0.69–0.95)	0.178
SPPB score, median (IQR)	9.0 (6.5–11.0)	10.5 (9.0–12.0)	9.0 (6.0–11.0)	0.002	9.0 (7.0–11.0)	9 (6.2–11.0)	0.204	9.0 (6.0–11.0)	10.0 (7.0–11.0)	0.128
ADL score > 5, n (%)	110 (91.7%)	23 (95.8%)	87 (90.6%)	0.679	55 (90.2%)	55 (93.2%)	0.783	51 (89.5%)	59 (93.7%)	0.619
IADL score > 6, n (%)	44 (36.7%)	13 (54.2%)	31 (32.3%)	0.079*	28 (45.9%)	16 (27.1%)	0.052*	26 (45.6%)	18 (28.6%)	0.081*
MMSE score, median (IQR)	24.0 (22.0–26.0)	25.0 (23.0–26.0)	24.0 (21.0–26.2)	0.213	24.0 (22.0–26.0)	25.0 (22.0–26.0)	0.736	23.0 (22.0–26.0)	25.0 (22.0–26.0)	0.516
CDR score (0.5 vs 1), n (%)	102 (85.0%)	21 (87.5%)	81 (84.4%)	0.949	51 (83.6%)	51 (86.4%)	0.858	48 (84.2%)	54 (85.7%)	1.000
Polypharmacy, n (%)	66 (57.9%)	10 (45.5%)	56 (60.9%)	0.282	33 (54.1%)	33 (57.9%)	1.000	30 (56.6%)	36 (59.0%)	0.944

*ADL* Activities of Daily Living, *AUR* Appropriate Use Recommendations, *BMI* Body Mass Index, *CDR* Clinical Dementia Rating, *IADL* Instrumental Activities of Daily Living, *IQR* Interquartile Range, *MMSE* Mini Mental State Examination, *SPPB* Short Physical Performance Battery

^§^For the American and French AUR, the category “yes” refers to individuals who are potentially eligible after applying the exclusion criteria; however, a proportion of them would require evaluation through a collegial discussion before being fully cleared for treatment

## Discussion

To the best of our knowledge, this is the first study to apply Lecanemab eligibility criteria – based on both the Clarity-AD trial and real-world recommendations from the American and French AUR—to a real-world cohort of pre-frail and frail individuals with objective cognitive impairment and confirmed brain amyloid pathology. Our findings show that while less than one in five participants from the Cogfrail cohort would have been eligible for inclusion in the Clarity-AD trial, approximately half the cohort would be potentially treatable with Lecanemab according to real-world recommendations. Moreover, while fewer than 10% of frail individuals would have been eligible for inclusion in the Clarity-AD trial, approximately 50% would potentially meet eligibility criteria for treatment based on real-world recommendations. In other words, nearly 80% of frail patients who could be potentially treated in clinical practice would not have been enrolled in the trial population, underscoring a potential gap between clinical trials and real-world practice, with interesting implications for the generalizability of Lecanemab’s efficacy and safety.

Previous studies applicating the phase III Clarity-AD trial to community-dwelling older adults with MCI or early dementia reported an eligibility rate ranging from 1.5% to approximately 30% [14–17]. The substantial variability across investigations primarily arises from differences in population selection criteria, making it difficult to compare eligibility rates across studies. For instance, some studies estimated eligibility rates exclusively among amyloid-positive individuals [14], while others initially included amyloid-negative subjects [15–17], only to exclude them later based on Clarity-AD eligibility criteria. A similar issue arose with AD dementia severity, as some studies initially included individuals with more advanced dementia (CDR > 1) [14, 16], only to exclude them later based on the trial's eligibility requirements. Considering that we estimated eligibility rates only among amyloid-positive individuals and that our cohort was preselected for the Cogfrail study—requiring participants to have a CDR score of 0.5 or 1—the eligibility rate observed in our sample may have been expected to be higher than that observed in previous studies. However, applying Clarity-AD criteria this was not the case, most likely due to the characteristics of our population (pre-frail and frail individuals), which may have influenced the eligibility rate.

Although phase III studies represent the cornerstone for ensuring the safety and efficacy of experimental drugs, real-world data are absolutely needed to confirm the generalizability of randomized controlled trial findings in populations that are more difficult to include in clinical trials. The discrepancy in eligibility rates between the Clarity-AD trial and real-world recommendations primarily reflects differences in inclusion criteria, which allowed for the potential inclusion of approximately one-fourth of participants who would have been excluded from the trial based solely on age, BMI, or MMSE scores. For instance, while an MMSE score ≥ 22 was a strict eligibility criterion in the Clarity-AD trial, real-world recommendations place greater emphasis on functional status, allowing for the inclusion of individuals with lower MMSE scores but a CDR compatible with MCI or mild dementia. Moreover, the application of clinical history and medication-related exclusion criteria appears to rely more on clinical judgment within the context of real-world recommendations, except for anticoagulation. This may allow for the inclusion—following appropriate management—of individuals who would have been excluded from the Clarity-AD trial due to factors such as uncontrolled diabetes or recent initiation of antidepressant therapy. In both the trial and the real-world recommendations, nearly one third of participants would have ultimately been excluded due to MRI findings, making this the leading cause of ineligibility for Lecanemab treatment in our sample. Although ApoE genotype data were missing for eight participants, our findings suggest that the ε4/ε4 genotype would have had minimal impact on eligibility decisions among pre-frail and frail older adults. The American and French recommendations demonstrated substantial concordance, with discrepancies in eligibility observed in only four cases.

Recent observational data suggest that approximately one in four individuals visited at Memory Clinics exhibits at least moderate frailty, regardless of whether the service is managed by neurologists or geriatricians [21]. This is particularly relevant, as our findings suggest that frail individuals could represent a substantial proportion of the patients potentially eligible for treatment in real-life clinical practice. This proportion may be even higher when applying drug label recommendations instead of the AUR (~ 60% of our population eligible under the EMA label criteria). However, although Clarity-AD did not assess frailty status, the relatively low proportion of frail individuals meeting the trial’s inclusion criteria in our cohort indirectly suggest that frailty may have been underrepresented in the trial, raising concerns about the generalizability of the outcomes in this population. Notably, the trial population was approximately 11 years younger than the median age of our cohort, and multimorbidity represented a major reason for exclusion in Clarity-AD. While frailty is not a direct consequence of either chronological age or multimorbidity, both are strongly associated with frailty in older adults. Taken together, these factors suggest that age and multimorbidity may have acted as proxies for frailty in Clarity-AD, contributing to the exclusion of frail individuals despite the absence of a formal frailty assessment. From a safety perspective, frailty is well recognized as a risk factor for increased susceptibility to adverse drug reactions (ADRs) [22]. Given that frailty reflects a state of heightened vulnerability to stressors, the response to pharmacological treatments may be different in this population [22]. Consequently, frail individuals may face a higher risk of experiencing ADRs, including infusion-related reactions, ARIAs, headaches, and falls. From an efficacy perspective, as highlighted by Wallace et al. [23], frail people may present a weaker relationship between AD pathology and Alzheimer’s dementia. In other words, frailty may diminish an individual's ability to tolerate AD pathology, potentially leading to cognitive decline even at low amyloid burdens that might have remained asymptomatic in non-frail individuals. It is indeed possible that without a more comprehensive and personalized approach, amyloid reduction alone may not yield meaningful clinical benefits. Frailty has long been used as a reason for treatment exclusion [24], but a more effective strategy should provide access to personalized treatment according to frailty status. This approach could involve combining pharmacological therapy (i.e., Lecanemab) with tailored interventions designed to enhance resilience, potentially increasing tolerability to residual brain pathology and reducing ADRs. Prehabilitation, a rehabilitation program designed to optimize function and improve tolerability to intensive medical interventions (i.e. surgery or chemotherapy), has become the standard of care in several medical and surgical fields [25]. In this context, a multidomain intervention aimed at improving frailty before and alongside anti-amyloid treatment may enhance treatment tolerability and potentially increase individuals' ability to cope with the residual AD-related brain pathology. Future research is needed to assess whether combining anti-amyloid treatment with multidomain interventions yields better outcomes than anti-amyloid therapy alone, particularly in frail individuals.

### Limitations

The study also some limitations. First, it is a secondary analysis of data originally collected for the Cogfrail study, whose primary objective differed from the current research question. Second, eligibility was determined through retrospective chart review. Third, although the sample is highly representative of a real-world population with objectively measured cognitive impairment, inevitably, there is also an overlap between Lecanemab and Cogfrail eligibility criteria, which may have contributed to overestimate the proportion of eligible individuals. Fourth, because frailty in our study was assessed using the Fried frailty phenotype, it is possible that alternative approaches to frailty measurement (e.g., the deficit accumulation model) might have yielded slightly different results. However, the study has also several strengths. The Cogfrail study includes neuroimaging and a comprehensive geriatric assessment for all participants, thereby enabling an accurate application of the eligibility criteria. Additionally, patients were recruited from outpatient visits at Frailty or Memory Clinics as part of their routine assessments, making the sample representative of real-world populations.

## Conclusions

In conclusion, although less than one-fifth of the Cogfrail cohort would have been included in the Clarity-AD trial, more than half would meet Lecanemab eligibility criteria according to both the American and the French recommendations. While a considerable proportion of frail patients may have access to Lecanemab treatment in real-life, the low proportion of potentially eligible frail individuals in our cohort indirectly suggests that frailty may have been underrepresented in the Clarity-AD trial, raising concerns about the generalizability of its findings to this population. Caution is warranted when targeting amyloid burden without addressing underlying frailty, as the risk–benefit ratio in this population may differ from that observed in the Clarity-AD study.

## Supplementary Information

Supplementary Material 1.

Supplementary Material 2.

## Data Availability

De-identified data from the COGFRAIL cohort are available to researchers upon request, following approval of a methodologically sound research proposal and signature of a data use agreement. Enquiries or proposals should be addressed to [sourdet.s@chu-toulouse.fr].

## Abbreviations

AD: Alzheimer’s disease
ADL: Activities of Daily Living
ApoE: Apolipoprotein E
ARIA-E: Amyloid-Related Imaging Abnormalities Edema
ARIA-H: Amyloid-Related Imaging Abnormalities Hemorrhages
AUR: Appropriate Use Recommendations
BMI: Body Mass Index
CAA: Cerebral Amyloid Angiopathy
CDR: Clinical Dementia Rating
CDR-SB: Clinical Dementia Rating Sum of Boxes
CLEIA: Chemiluminescent Enzyme Immunoassay
Cogfrail: Cognitive Function and Amyloid Marker in Frail Older Adults
CRB: Centre de Ressources Biologiques
CSF: Cerebrospinal Fluid
DMT: Disease-Modifying Therapy
ELISA: Enzyme-Linked Immunosorbent Assay
EMA: European Medicines Agency
ICD: Implantable Cardioverter-Defibrillator
IQR: Interquartile Range
MMSE: Mini-Mental State Examination
MRI: Magnetic Resonance Imaging
PET: Positron Emission Tomography
SPPB: Short Physical Performance Battery
