# Supplementary material for: Are pre-frail and frail amyloid positive individuals eligible to Lecanemab? A cross-sectional analysis from the Cogfrail real-world cohort
Source: Alzheimers Res Ther. 2026 Feb 4;18:56. doi: 10.1186/s13195-026-01966-0 (PMC12964647; doi:10.1186/s13195-026-01966-0)
Supplement: Supplementary file 1 — Supplementary Material 1. [file 13195_2026_1966_MOESM1_ESM.docx]

**Supplementary Table 1.** Differences between the Clarity-AD eligibility criteria and the American and European recommendations for appropriate Lecanemab use. Differences from the Clarity-AD eligibility criteria are highlighted in red.

|  | CLARITY-AD Criteria | American AUR | French AUR |
| --- | --- | --- | --- |
| Inclusion Criteria | | | |
| Cognitive severity | MMSE score ≥ 22 and ≤ 30 at baseline visits | MMSE ≥ 22 or another cognitive test compatible with early AD (e.g., CDR 0.5-1) | MMSE ≥ 22 can be discussed in special situations (e.g., low educational level or language difficulties) in the context of early AD |
| Age | Between 50 and 90 years old | Clinical judgment for individuals younger than 50 or older than 90 years | No exclusions based only on chronological age |
| BMI | BMI between 17 and 35 at screening | Clinical judgment for individuals with a BMI below 17 or above 35 | Clinical judgment for individuals with a BMI below 17 or above 35 |
| Exclusion criteria | | | |
| ApoE4 status | No exclusions based on ApoE4 status | Clinical judgment for ApoE4 status-based exclusions | Not recommended for APOE4 homozygosis  Additional MRI monitoring for ARIA in APOE4 heterozygosis |
| Anticoagulants | Eligible if on a stable dose for at least four weeks | Not recommended for patients on anticoagulants | Not recommended for patients on anticoagulants |
| Brain MRI | Brain MRI at screening visit | Brain MRI within 6 months prior to Lecanemab administration | Brain MRI within 6 months prior to Lecanemab administration |
|  | Infarcts or stroke involving a major vascular territory | Stroke involving a major vascular territory | Stroke affecting a major vascular territory should be assessed individually |
| CAA | Not specifically addressed: “More than four micro-hemorrhages” | Not specifically addressed: “More than four micro-hemorrhages” | People with CAA should be excluded from Lecanemab treatment: More than four micro-hemorrhages (lobar and/or deep) or more than one lobar microhemorrhage (probable CAA) |
| Neurological comorbidities | Any neurological disorder potentially contributing to cognitive decline | Any neurological disorder potentially contributing to cognitive decline | Interdisciplinary meetings |
| Depression | GDS score greater than 8 at screening | Severe major depression | Interdisciplinary meetings |
| Other comorbidities | Any medically unstable conditions | Any medically unstable conditions that could impact or be impacted by treatment | Interdisciplinary meetings |

Legend: AD, Alzheimer’s Disease; BMI, Body Mass Index; CAA, Cerebral Amyloid Angiopathy; CDR, Clinical Dementia Rating; GDS, Geriatric Depression Scale; MMSE, Mini Mental State Examination; MRI, Magnetic Resonance Imaging.
