# Supplementary material for: Are pre-frail and frail amyloid positive individuals eligible to Lecanemab? A cross-sectional analysis from the Cogfrail real-world cohort
Source: Alzheimers Res Ther. 2026 Feb 4;18:56. doi: 10.1186/s13195-026-01966-0 (PMC12964647; doi:10.1186/s13195-026-01966-0)
Supplement: Supplementary file 2 — Supplementary Material 2. [file 13195_2026_1966_MOESM2_ESM.docx]

**Supplementary Table 2.** Reasons for exclusion due to relevant clinical history in the Clarity-AD trial (n = 25) and reasons for discussion in multidisciplinary meetings according to the American and French AUR (n = 18 and n = 15, respectively).

|  | Clarity AD exclusion for relevant clinical history | Multidisciplinary meetings | |
| --- | --- | --- | --- |
| Conditions |  | American AUR | French  AUR |
| Uncontrolled depressive symptoms, n (%) | 6 (24.0%) | 5 (27.8%) | 5 (33.3%) |
| Sequelae of cerebrovascular stroke, n (%) | 5 (20.0%) | / | / |
| Malignancy treated with CT within the past 3 years, n (%) | 3 (12.0%) | 2 (11.0%) | 2 (13.3%) |
| Uncontrolled hypothyroidism, n (%) | 2 (8.0%) | / | / |
| Severe chronic kidney disease, n (%) | 2 (8.0%) | / | / |
| Meningioma, n (%) | 2 (8.0%) | 1 (5.6%) | 1 (6.7%) |
| Uncontrolled type 2 diabetes mellitus, n (%) | 1 (4.0%) | 1 (5.6%) | 1 (6.7%) |
| Symptomatic peripheral arterial occlusive disease, n (%) | 1 (4.0%) | 1 (5.6%) | 1 (6.7%) |
| Uncontrolled behavioral disturbances, n (%) | 1 (4.0%) | 1 (5.6%) | 1 (6.7%) |
| Uncontrolled arterial hypertension, n (%) | 1 (4.0%) | 1 (5.6%) | 1 (6.7%) |
| Hyperthyroidism, n (%) | 1 (4.0%) | / | / |
| Heart failure with reduced ejection fraction (HFrEF), n (%) | 1 (4.0%) | / | / |
| Immunosuppressive treatment, n (%) | / | 2 (11.0%) | 2 (13.3%) |
| Post–hepatitis B virus–related cirrhosis, n (%) | / | 1 (5.6%) | 1 (6.7%) |
| Age > 90 years, n (%) | Excluded for inclusion criteria | 3 (16.6%) | / |
| Total | 25 | 18 | 15 |
